# Congenital Toxoplasmosis With Atypical Neuroimaging: Hydrocephalus and Bilateral Chorioretinitis Without Intracranial Calcifications

**DOI:** 10.1155/crra/3021918

**Published:** 2026-04-02

**Authors:** Saubhagya Dhakal, Pradeep Raj Regmi, Prajwal Dhakal, Binaya Adhikari, Shashi Shekhar Shingh, Basanta Sharma Paudel

**Affiliations:** ^1^ Department of Radiology and Imaging, Bir Hospital, National Academy of Medical Sciences, Kathmandu, Nepal, nams.org.np; ^2^ Department of Radiology and Imaging, Tribhuvan University Teaching Hospital, Kathmandu, Nepal, teachinghospital.org.np

**Keywords:** chorioretinitis, congenital toxoplasmosis, hydrocephalus, neuroimaging, prenatal care, resource-limited setting

## Abstract

We report a rare case of congenital toxoplasmosis in a 2‐month‐old female infant from rural Nepal, born to a primigravida mother without prenatal care. The infant presented with progressive lethargy, feeding difficulties, intermittent fever, and progressive macrocephaly. Neuroimaging revealed obstructive hydrocephalus and multiple cerebral ring‐enhancing lesions without intracranial calcifications. Ophthalmologic examination demonstrated bilateral chorioretinitis. Serologic testing confirmed congenital toxoplasmosis. Despite initiation of standard therapy with pyrimethamine, sulfadiazine, and folinic acid, the infant experienced clinical deterioration prior to planned neurosurgical intervention. This case highlights the clinical and radiologic variability of congenital toxoplasmosis, the diagnostic challenges in resource‐limited settings, and the importance of recognizing atypical imaging patterns to guide timely management.

## 1. Introduction

Congenital toxoplasmosis is caused by *Toxoplasma gondii* and is transmitted vertically from mother to fetus [1]. Its prevalence varies worldwide due to differences in dietary habits, environmental exposure, and socioeconomic factors [2, 3]. Globally, nearly one‐third of the population is infected, with congenital infection rates ranging from 1 to 14 per 10,000 live births depending on the region [3]. Although comprehensive data are limited in South Asia, congenital toxoplasmosis imposes a significant burden in countries such as Nepal, where it accounted for an estimated 9255 disability‐adjusted life years (DALYs) annually between 2000 and 2012 [4]. Seroprevalence studies indicate frequent maternal exposure, with antibodies detected in 55.4% of pregnant Nepalese women [5].

The gestational age at maternal infection influences clinical outcomes. Transmission during the first trimester, although less common, is associated with more severe manifestations [2, 6]. Approximately 75% of infected infants are asymptomatic, but some develop life‐threatening complications [2, 7, 8]. The classic triad—hydrocephalus, intracranial calcifications, and chorioretinitis—is now infrequently observed in its complete form [2]. Symptomatic cases may present with systemic signs (preterm birth, rash, and sepsis‐like illness), neurological features (microcephaly, hydrocephalus, seizures, hypotonia, and sensorineural hearing loss), and ocular involvement (cataract, chorioretinitis, nystagmus, strabismus, and microphthalmia) [9, 10]. These findings are nonspecific and overlap with other congenital infections such as cytomegalovirus, herpes simplex, rubella, or syphilis [2, 10].

Early diagnosis remains challenging, particularly in settings with limited access to diagnostic tools, and delayed recognition often results in irreversible neurological damage [11, 12]. The absence of universal prenatal screening in many countries further contributes to missed or late diagnoses, highlighting a significant public health gap.

This report describes a severe presentation of congenital toxoplasmosis in a 2‐month‐old infant from a resource‐limited setting, illustrating how limited prenatal care can lead to serious clinical consequences. We also demonstrate characteristic neuroimaging findings, including hydrocephalus, multiple ring‐enhancing lesions, and bilateral chorioretinitis without intracranial calcifications, emphasizing the educational value of recognizing atypical presentations.

## 2. Case Presentation

A 2‐month‐old female infant from rural Nepal was referred to our tertiary care hospital with progressive lethargy, poor feeding, intermittent fever, and increasing head circumference. The mother was a 23‐year‐old primigravida with no antenatal visits. During the second trimester, she reported a brief febrile illness with malaise that resolved without medical evaluation. No prenatal screening for *T. gondii* or other congenital infections had been performed.

The infant was delivered at term at home. Macrocephaly was not evident at birth, and the estimated head circumference was approximately 33 cm, within the normal range for term female neonates. At 2 months of age, the head circumference measured 41 cm (> 97th percentile), consistent with progressive hydrocephalus.

On Day 5 of life, the infant developed persistent jaundice lasting more than 1 month. At a local primary care center, she was treated with oral broad‐spectrum antibiotics for presumed neonatal sepsis, initiated due to intermittent fever, lethargy, and poor feeding. Intravenous therapy and advanced imaging were not available at that facility. Symptoms did not improve, and progressive head enlargement prompted referral.

At presentation, the infant appeared ill and lethargic, with dehydration and persistent jaundice. Physical examination revealed mild hepatosplenomegaly, with the liver palpable 2 cm below the right costal margin and the spleen just palpable. Neurologic examination showed axial hypotonia, poor suck reflex, diminished spontaneous movements, and delayed responsiveness. The anterior fontanelle was tense and bulging, with widened cranial sutures. Ophthalmologic evaluation demonstrated bilateral chorioretinitis with multiple necrotic retinal lesions.

Laboratory testing revealed anemia (hemoglobin 8.2 g/dL), thrombocytopenia (95,000/*μ*L), and elevated liver enzymes (AST 135 U/L, ALT 102 U/L). Serum bilirubin demonstrated conjugated hyperbilirubinemia (total 6.5 mg/dL; direct 4.1 mg/dL).

Cerebrospinal fluid was obtained via lumbar puncture at the L3–L4 interspace under aseptic conditions. CSF analysis showed elevated protein (160 mg/dL), low glucose (35 mg/dL), and lymphocytic pleocytosis (40 WBC/mm^3^).

Infant serology demonstrated markedly elevated anti‐*Toxoplasma* IgM of 6.8 units (reference ≥ 2.0 units) and IgG of 120 IU/mL (reference ≥ 15 IU/mL). Maternal serology showed negative IgM with elevated IgG, consistent with prior infection and vertical transmission. Screening for rubella, cytomegalovirus, and syphilis was negative.

**Figure 3:**
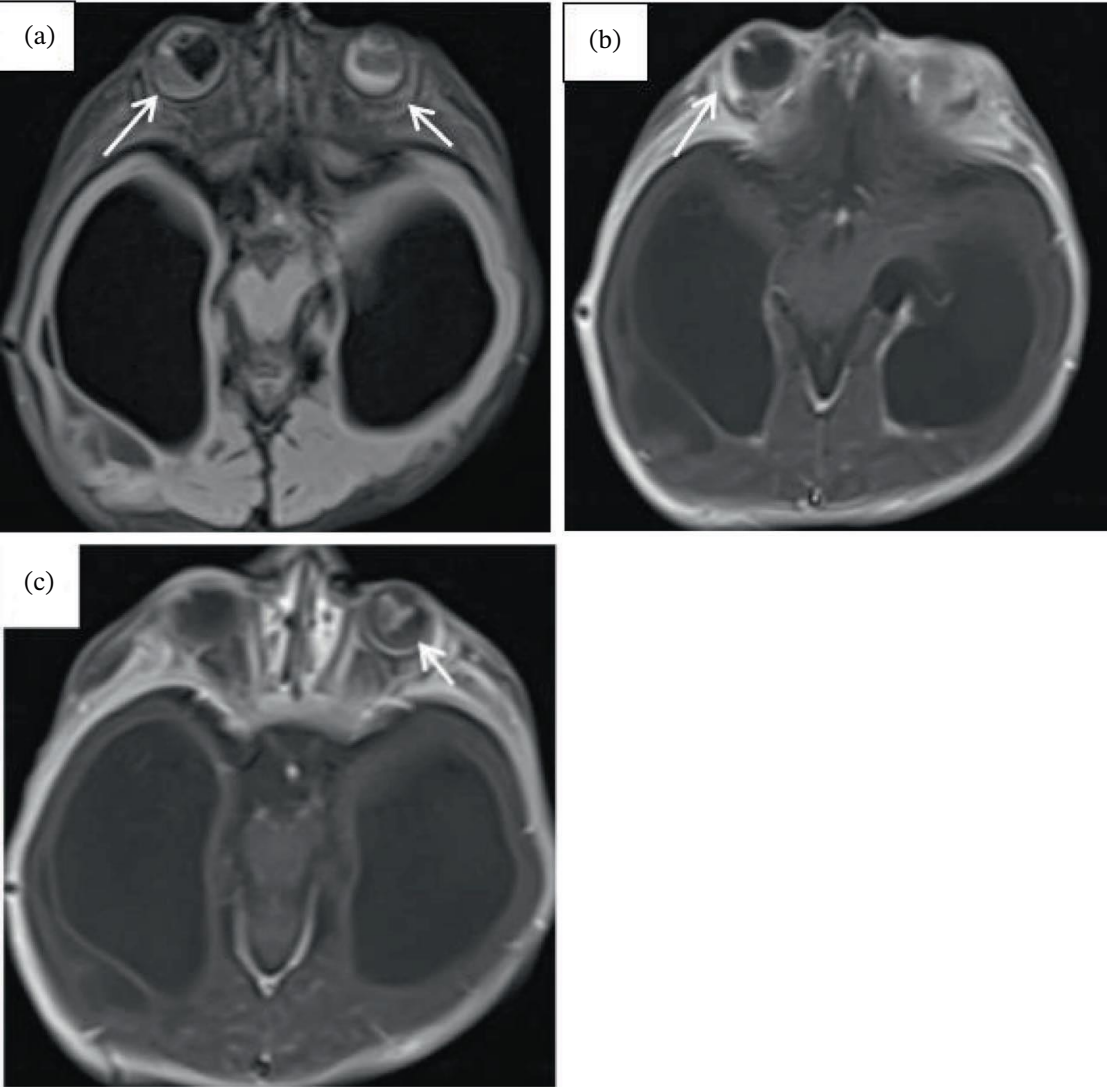
Bilateral chorioretinitis. (a) Axial FLAIR MRI demonstrates hyperintense signal involving the bilateral chorioretina. (b, c) Postcontrast fat‐suppressed axial T1‐weighted images show corresponding enhancing lesions.

Cranial ultrasound revealed marked ventriculomegaly. Brain MRI confirmed obstructive hydrocephalus with gross dilation of the lateral and third ventricles (Figure 1a, 1b). Postcontrast T1‐weighted images demonstrated multiple ring‐enhancing lesions in the right temporoparietal and left frontal subcortical regions (Figures 2a, 2b, and 2c). MRI did not demonstrate evidence of intracranial calcifications. Bilateral high signal intensity within the chorioretina on T2‐FLAIR sequences supported the diagnosis of chorioretinitis (Figures 3a, 3b, and 3c).

Figure 1Obstructive hydrocephalus on MRI. (a) Axial T2‐weighted MRI demonstrates marked dilatation of the lateral ventricles with transependymal edema. (b) Sagittal T1‐weighted MRI shows dilatation of the lateral and third ventricles with aqueductal obstruction.

**Figure:**
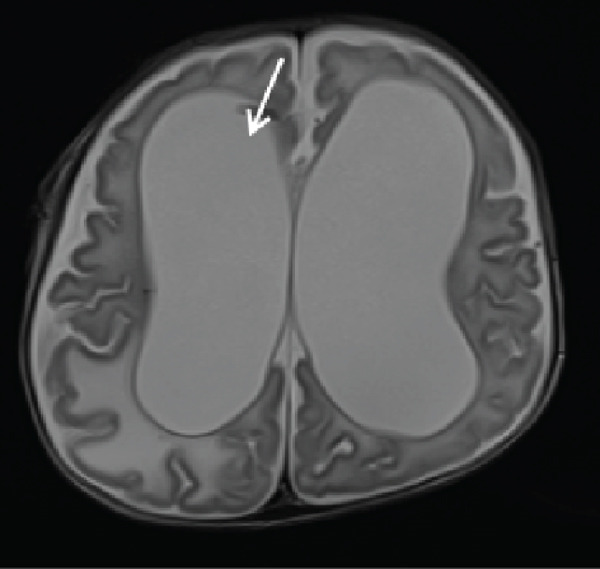
(a)

**Figure:**
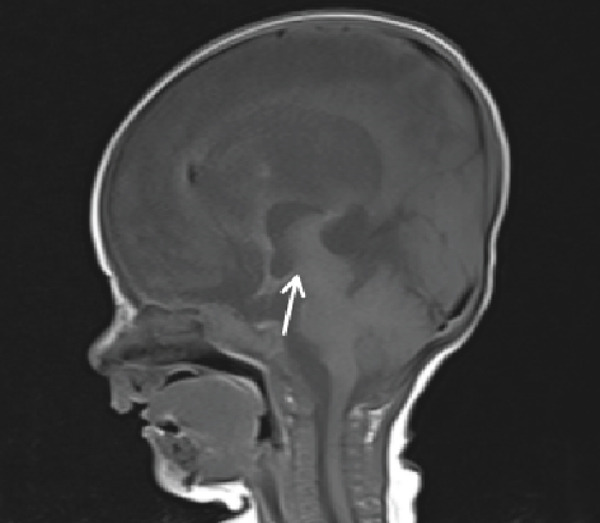
(b)

Figure 2Ring‐enhancing lesions.(a‐c) Postcontrast T1‐weighted MRI in axial and coronal planes demonstrates multiple enhancing lesions in the right temporoparietal region and left frontal lobe.

**Figure:**
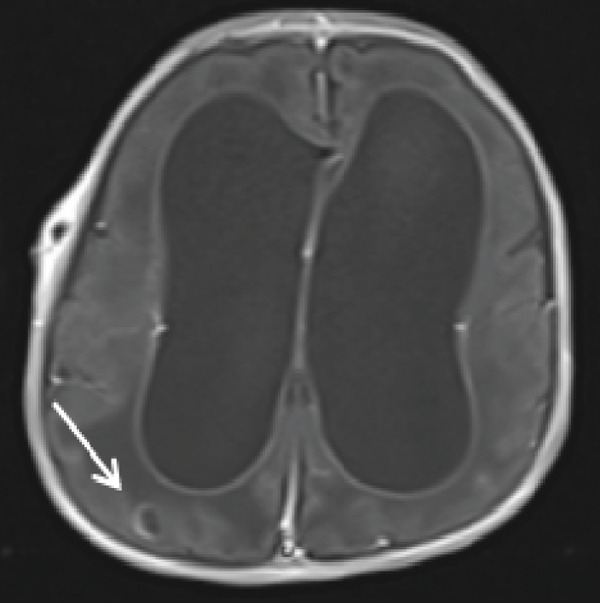
(a)

**Figure:**
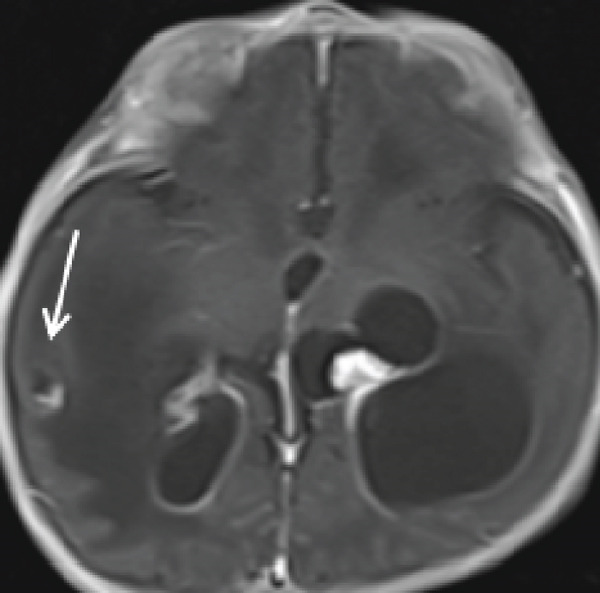
(b)

**Figure:**
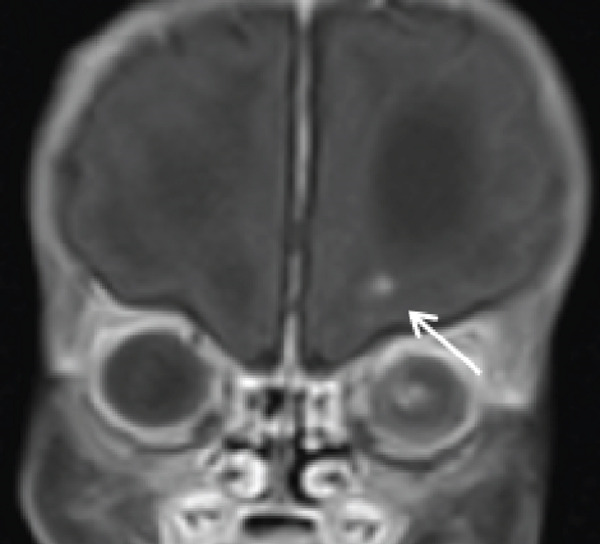
(c)

Treatment with pyrimethamine, sulfadiazine, and folinic acid was initiated. Neurosurgical consultation recommended ventriculoperitoneal shunt placement. Despite therapy, the infant experienced clinical deterioration prior to surgical intervention.

## 3. Discussion

Congenital toxoplasmosis demonstrates significant clinical and radiologic variability, influenced by the timing of maternal infection and host immune response [2, 6]. Although the classical triad of hydrocephalus, intracranial calcifications, and chorioretinitis is frequently described, it is now infrequently observed in its complete form [2]. Although hydrocephalus and periventricular calcifications remain commonly reported imaging findings [2, 9], atypical manifestations such as cerebral atrophy, periventricular white matter lesions, or ring‐enhancing lesions have also been reported [7, 9]. Recognition of this heterogeneity is critical, as reliance solely on classical descriptions may delay diagnosis.

In this case, obstructive hydrocephalus and bilateral chorioretinitis were present, but intracranial calcifications were not identified on MRI. Although calcification‐negative cases are less common, they have been documented in the literature [2, 9]. This underscores the importance of comprehensive neuroimaging assessment and cautious interpretation in suspected congenital infections.

Neuroimaging plays a central role in diagnosis and management. Although hydrocephalus, cerebral atrophy, and periventricular calcifications are often emphasized in textbooks, this patient demonstrated obstructive hydrocephalus with multiple ring‐enhancing lesions and bilateral chorioretinitis without calcifications, highlighting the variability of radiologic manifestations. Cranial ultrasound provided an accessible first‐line assessment by demonstrating ventriculomegaly, whereas MRI more precisely delineated ventricular dilatation, parenchymal lesions, and ocular involvement.

Differential diagnoses included congenital cytomegalovirus infection, rubella, and Zika virus infection [10, 13, 14], as well as noninfectious structural and genetic conditions such as L1 syndrome and Aicardi–Goutières syndrome [15, 16]. Structural midline anomalies, including corpus callosal agenesis and related interhemispheric cystic malformations, may present with ventriculomegaly and developmental delay and can mimic congenital infections on imaging [17, 18]. However, hallmark neuroradiologic findings such as complete or partial callosal agenesis, interhemispheric cysts, or characteristic callosal dysgenesis were not identified in this patient. Serologic confirmation of *T. gondii* infection supported congenital toxoplasmosis as the final diagnosis.

Early initiation of pyrimethamine, sulfadiazine, and folinic acid therapy is associated with improved neurodevelopmental outcomes [8, 19]. Surgical management of hydrocephalus, including ventriculoperitoneal shunt placement, may further improve outcomes in selected cases [2, 20]. In this patient, delayed recognition limited the opportunity for early intervention, underscoring the importance of timely imaging and prompt treatment.

This case highlights the educational value of recognizing atypical imaging features in congenital toxoplasmosis. Radiologists should consider the diagnosis even in the absence of calcifications when hydrocephalus, ring‐enhancing lesions, and ocular involvement are present. Awareness of calcification‐negative presentations enhances diagnostic accuracy and supports appropriate management, particularly in resource‐limited settings.

## 4. Limitations

This case has several limitations. Cranial CT was not performed, which may have limited detection of subtle intracranial calcifications, although MRI provided sufficient diagnostic information while avoiding radiation exposure. Long‐term neurodevelopmental follow‐up was unavailable, restricting outcome assessment. Additionally, the timing of maternal infection could not be determined due to the absence of prenatal screening and serial serologic evaluation.

## 5. Conclusion

This case illustrates an atypical presentation of congenital toxoplasmosis characterized by obstructive hydrocephalus, multiple ring‐enhancing lesions, and bilateral chorioretinitis without intracranial calcifications. The absence of intracranial calcifications does not exclude the diagnosis. Awareness of the clinical and radiologic variability of congenital toxoplasmosis is essential, particularly in resource‐limited settings, where delayed recognition may adversely affect outcomes. Early imaging and prompt treatment remain critical for improving neonatal prognosis.

## Funding

No funding was received for this manuscript.

## Ethics Statement

Written informed consent was obtained from the patient′s parents for publication of this case report and accompanying images. Institutional review board approval was not required for a single case report in accordance with local policy.

## Conflicts of Interest

The authors declare no conflicts of interest.

## Data Availability

The data that support the findings of this study are available from the corresponding author upon reasonable request.
